# A severe presentation of breakthrough infection caused by the Omicron variant with radiological findings of COVID-19 pneumonia in an elderly woman

**DOI:** 10.1016/j.radcr.2022.06.034

**Published:** 2022-07-12

**Authors:** Barbara Brogna, Chiara Capasso, Giovanni Fontanella, Elio Bignardi

**Affiliations:** a Department of Radiology, San Giuseppe Moscati Hospital, Contrada Amoretta, 83100 Avellino, Italy; b Pharmacology Department, “Frangipane” Hospital, ASL Avellino, Via V. Emanuele, 83031 Ariano Irpino, Italy; c Department of Radiology, Sacred Hear of Jesus Hospital- Fatebenefratelli, 82100, Benevento, Italy; d Radiology Unit, “Cotugno Hospital”, Naples, Via Quagliariello 54, 80131 Naples, Italy

**Keywords:** COVID-19, Elderly, Omicron variant, Immunosenescence, Vaccines

## Abstract

Omicron variant of COVID-19 is characterized by exceptional transmissibility and by immune evasion with the ability infect people with naturally acquired or vaccine-induced immunity. However, lung involvement is poorly reported in patients who resulted positive by this new COVID-19 variant. COVID-19 breakthrough infections are defined as COVID-19 infection in fully vaccinated patients. Herein, we present a case of breakthrough infection in an elderly woman who came in emergency with dyspnea and with findings of COVID-19 pneumonia on chest computed tomography. The patient was vaccinated with a booster dose of an mRNA vaccine some months earlier and the Omicron variant was detected on real-time reverse-transcription polymerase chain reaction. However, the patient's condition remained stable. For our knowledge we report the first case with lung involvement due to Omicron variant in an elderly after the booster dose of mRNA vaccine. This case highlights as COVID-19 breakthrough infections may represent some concerns in the elderly patients in presence of virus variants.

## Introduction

The coronavirus disease 2019 (COVID-19) pandemic caused by the severe acute respiratory syndrome coronavirus (SARS-CoV-2) has caused millions of deaths worldwide. Poor outcomes are especially observed in elderly people and in those with comorbidities [1,2].

COVID-19 presentation may vary from asymptomatic forms to a multisystem disease with the involvement of the lungs, gastrointestinal system, and central nervous system. COVID-19 can have later manifest as long COVID [1], [2], [3], [4].

COVID-19 vaccines represent an efficient strategy to control the spread and severity pandemic. COVID-19 vaccines have shown high efficacy in preventing severe disease in clinical trials [5,6]. Nevertheless, variants of COVID-19 have been cause for some concern [2,[7], [8], [9]. The emerging B.1.1.529 (Omicron) variant is characterized by multiple mutations, rapid spreading, and the ability infect people with naturally acquired or vaccine-induced immunity [8,9]. Therefore, the Omicron variant is more likely to escape vaccine-induced immune protection.

Recent data have indicated that B.1.1.529 is more transmissible than previous variants due to its large number of additional mutations in the spike protein [8], [9], [10]. However, the Omicron variant less frequently causes severe disease than the previous variants [10].

The efficacy of the COVID-19 vaccines should be enhanced given the decline in the immune response over the time, mainly in the elderly, in patients with comorbidities, and in those who are immunocompromised as such patients are also at major risk of developing severe disease [11], [12], [13], [14], [15]

We report a case of COVID-19 pneumonia due to the Omicron variant in an elderly woman who had previously received a third dose of an mRNA vaccine.

## Case presentation

A 75-year-old woman with a history of arterial hypertension and chronic renal failure was vaccinated with 3 doses of COVID-19 mRNA (the first one in early April of 2021, the second one was received 3 weeks later, and the booster at the end of September 2021).

**Fig. 1:**
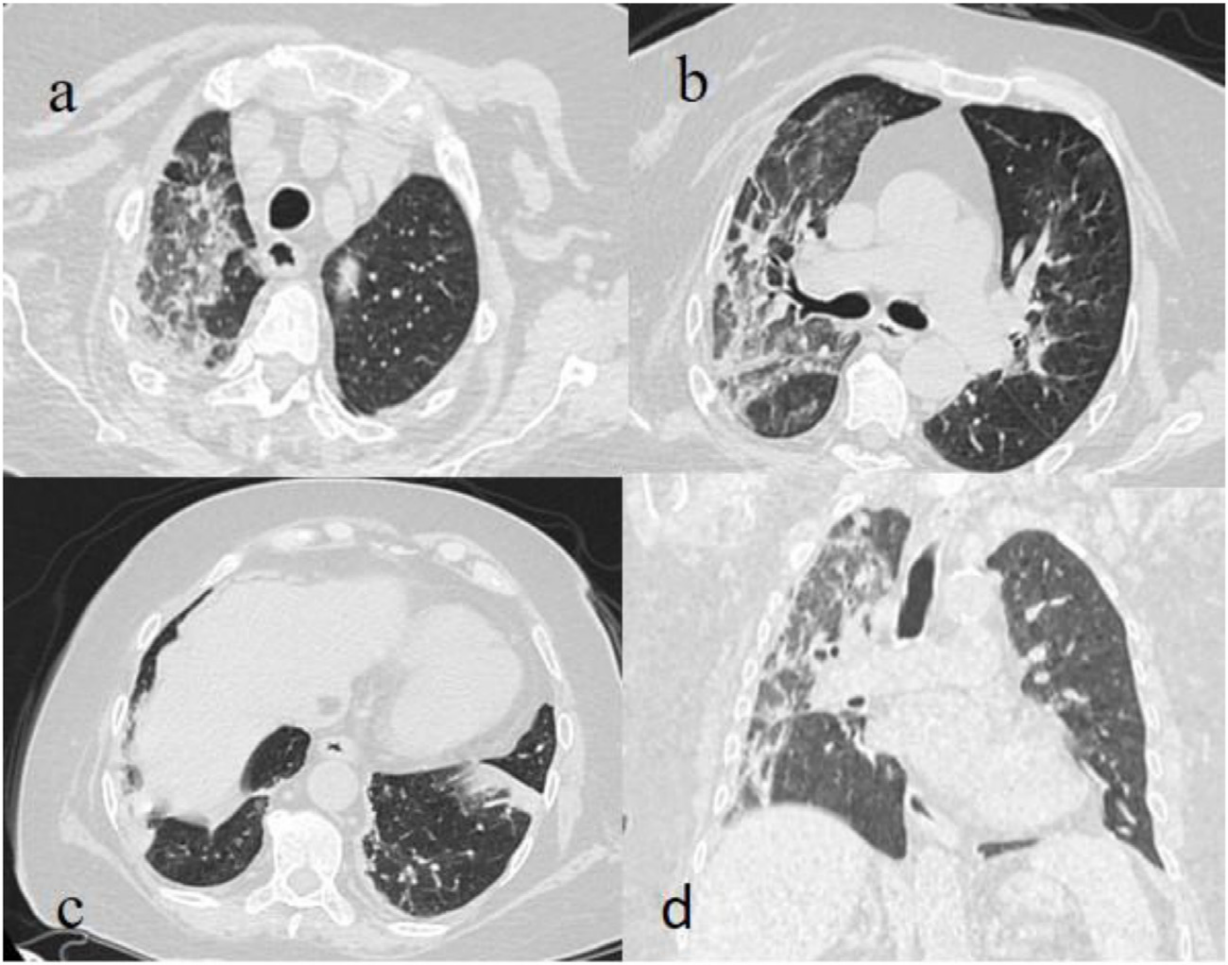
Chest CT performed at the baseline. In the images “a” and “b”, and “c” is represented the COVID-19 pneumonia with interstitial thickness with GGOs and initial consolidations in a multifocal distributions in the superior and inferior lobes with a predominance on the right side on the axial plane, in the image “d” on the coronal plane.

However, in the first days of December, she begun to experience fever (39°C) with cough and dyspnea with positive results on a real-time reverse-transcription polymerase chain reaction test for SARS-CoV-2 on nasopharyngeal/oropharyngeal (NP/OP) swabs, which detected the B.1.1.529 (Omicron) variant. One week later, her condition worsened with a blood oxygen saturation level (SO2) of 92%. Upon laboratory examination, she had a high white blood count (13.64 × 10^3^/µL), elevated lactated dehydrogenase enzyme (465 U/L), elevation of C-reactive protein (16.6 mg/dL), and a mild elevation in the D-dimer level (367 ng/mL). A chest computed tomography (CT) was also conducted in the emergency room. The chest CT showed interstitial thickness with ground glass opacities and initial consolidations suggesting COVID-19 pneumonia, with a score of 13/20 (Fig. 1).

The patient received treatment with dexamethasone (4 mg once daily with intravenous administration for 10 days) along with conventional oxygen therapy, which was started together with LMWE 4000 IU (twice daily with subcutaneous administration for 10 days). The serology, performed with an immunoassay (Liaison XL), showed only the presence of SARS‐CoV‐2 anti‐spike IgG (>2080 BAU/mL) related to the previous vaccination (<33.80 BAU/mL: absent; >33.80 BAU/mL: presence).

**Fig. 2:**
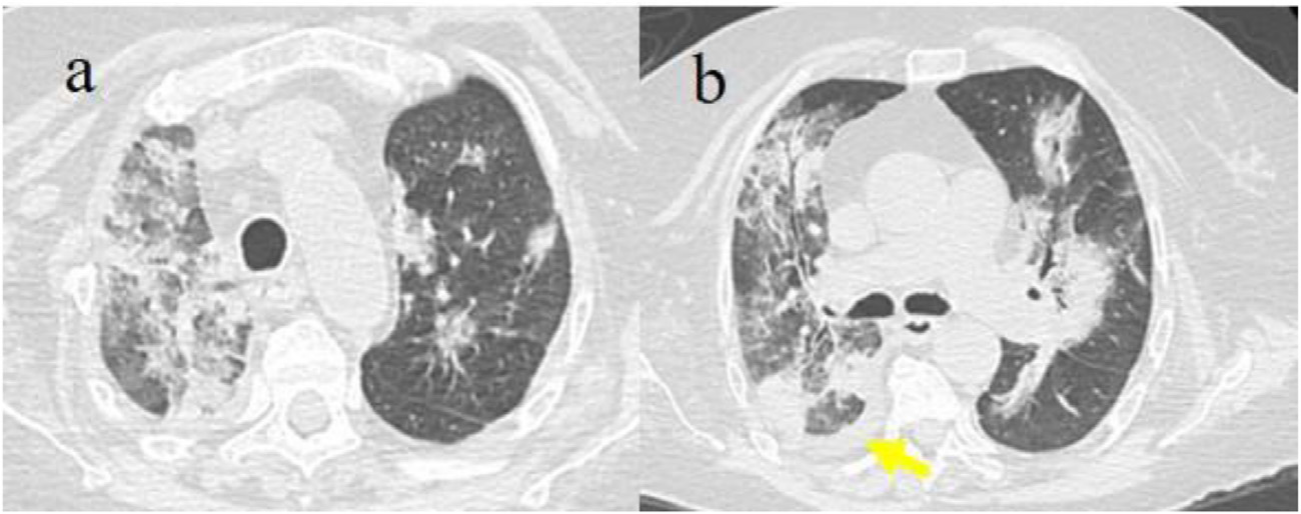
Chest-CT performed 1 week later. In the image “a” and “b” is represented a more pronounced consolidations of the COVID-19 pneumonia in the superior lobes with a mild pleural effusion on the right side (yellow arrow).

One week later, the patient showed a clinical exacerbation of the dyspnea with SO2 of 89%. On the chest CT was reported a mild worsening of the previous pneumonia with a CT-SS of 16/20 (Fig. 2). A mild pleural effusion was also found on the right side.

**Fig. 3:**
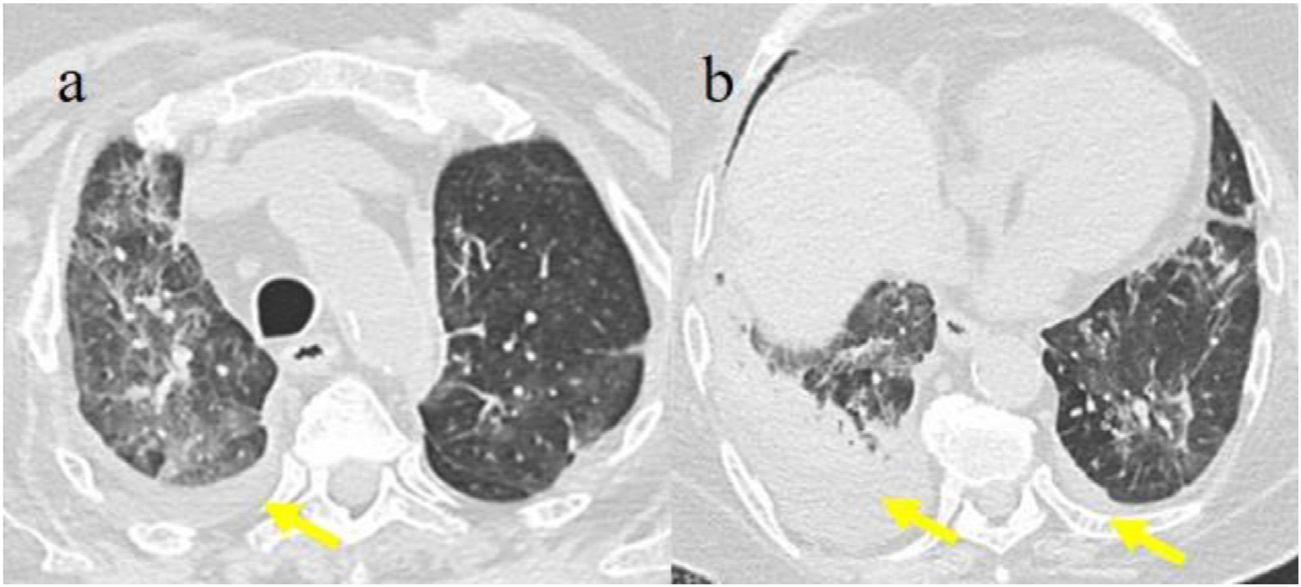
Chest CT performed after other 10 days that showed a mild reduction of the consolidations areas with increased appearance of the right pleural effusion (yellow arrow in the image a and b) and a small pleural effusion also on the left side (yellow arrow in the image b).

However, after other 10 days the patient showed a mild improvement of the dyspnea with SO2 of 95%. Another chest CT was repeated with improvement of the consolidation areas and with bilateral pleural effusion that was found increased on the right side (Fig. 3).

The patient's clinical conditions remained stable and she reported the first negative results for SARS-Cov-2 on NP/OP swabs after 30 days from the hospital admission. Therefore, the patient was transferred to another Hospital where she was followed for the lung rehabilitation for other 20 days.

## Discussion

COVID-19 pneumonia was usually reported on imaging during the first and second waves of the pandemic when COVID-19 vaccines were not available.

A COVID-19 vaccine breakthrough infection is defined as the detection of SARS-CoV-2 in a respiratory specimen collected from an individual who is fully vaccinated and in which the SARS-CoC-2 infection usually manifests with mild symptoms. However, severe presentations are also possible mainly in the elderly, in patients with comorbidities, and in those who are immunocompromised [11,[13], [14], [15]. This has triggered calls to intensify vaccination programs including vaccine booster doses [16]. The efficacy of a third booster of vaccines was evidenced in data from Israel [16,17]; this third dose is used to restore the immune response due to its decline in efficacy over time [16], [17], [18], [19]. Barda et al [17] showed that the third dose was effective in protecting individuals against severe COVID-19-related outcomes compared with receiving only two doses at least 5 months ago.

The Omicron variant is characterized by immune evasion and increased incidence of reinfections and breakthrough infections [19], [20], [21]. Some studies [16,22] confirmed that COVID-19 vaccines continued to be less effective against symptomatic infection with the Omicron compared with the Delta variant. Despite the previous variants of SARS-CoV-2 infection that predominantly affected the lower respiratory tract, the Omicron variant seem to predominantly affect the upper airways and can cause acute laryngitis and epiglottitis [23]. The danger of hospitalization or death were respectively the 59% and 69% lower in patients with Omicron infection compared to those infected with the Delta variant [24].

On the other hand, the study of Hui et al [25] showed that Omicron replicates faster than all other SARS-CoV-2 variants studied in the bronchi but less efficiently in the lung parenchyma and this finding explained the reduce reduced severity of Omicron.

However, in our case, the patient developed COVID-19 pneumonia 3 months after the third dose and was infected by the Omicron variant. In our patient the breakthrough infection can be also explained by the decline in the immune response over time after a booster dose of COVID-19 vaccines [16,22]. Nevertheless, it should be taken into account that the immunosenescence in the elderly can decrease the humoral response leading in the reduction of vaccine efficacy [26]. In our case, the patient showed also pleural effusion that is an atypical finding in COVID-19 pneumonia and reported mainly in critical ill patients in the previous waves of pandemic [27].

For our knowledge, we report the first case with lung involvement due to Omicron variant in an elderly after the booster dose of mRNA vaccine.

Imaging continues to play an important role and should be performed for patients at high risk of severe breakthrough infections [14,15].

This case highlights that particular attention should be paid to the elderly and patients with comorbidities, even if they were fully vaccinated especially in the presence of virus variants. We should also consider that vaccines are based only on the spike protein; therefore, virus variants may continue to pose some risk [28,29].

New vaccines that are not only based on spike proteins and produce a protective immune response that declines more slowly over time should be taken in account [28]. Furthermore, continued updates of COVID-19 vaccines should be considered [28,29].

Data regarding nasal spray vaccines that can block SARS-CoV-2 transmission are also promising [30] and they can induce also mucosal immunity.

On the other hand, early therapy in vaccinated and fragile individuals should be not overlooked after the third vaccine dose. The use of masks in all closed environments should be required in these individuals [16,22].

In conclusion, COVID-19 knowledge is continuously evolving and virus variants can represent some challenges in the elderly people. Therefore, a multilevel and integrated approach is necessary in order to manage COVID-19 patients. Vaccines updating and improvement are required.

## Institutional review board statement

The authors comply with international and national ethical standards. The study was conducted in accordance with the Declaration of Helsinki.

## CRediT authorship contribution statement

**Barbara Brogna:** Conceptualization, Methodology, Software, Validation, Formal analysis, Writing – original draft, Project administration. **Chiara Capasso:** Validation, Formal analysis. **Giovanni Fontanella:** Software, Formal analysis. **Elio Bignardi:** Conceptualization, Methodology, Software, Validation, Formal analysis, Writing – review & editing, Project administration.
